# Over than three-year follow-up results of thermal ablation for papillary thyroid carcinoma: A systematic review and meta-analysis

**DOI:** 10.3389/fendo.2022.971038

**Published:** 2022-10-24

**Authors:** JiaNan Xue, DengKe Teng, Hui Wang

**Affiliations:** Department of Ultrasound, China-Japan Union Hospital, Jilin University, Changchun, China

**Keywords:** thermal ablation, papillary thyroid carcinoma, long-term efficacy, systematic review and meta-analysis, MWA, RFA

## Abstract

**Background:**

This study is a meta-analysis based on evidence-based medicine to explore the long-term (≥3 years) efficacy of thermal ablation in the treatment of papillary thyroid carcinoma (PTC).

**Methods:**

We searched the PubMed, Embase, and Cochrane Library databases for studies published during the time between the establishment of the databases through June 2022. We included 13 non-randomized-controlled trials (non-RCTs) that reported the application of ultrasound-guided thermal ablation in PTC. We excluded studies that were repeated publications, research without full text, contained incomplete information, lacked data extraction, involved animal experiments, reviews, and systematic reviews. STATA 15.1 software was used to analyze the data.

**Results:**

Tumor volume after thermal ablation at 3-year follow-up was significantly lower than pre-ablation (standardized mean difference [SMD] = -1.06, 95% CI: -1.32~-0.80). The pooled results indicated that the maximum diameter after thermal ablation at 3-year follow-up was significantly lower than pre-ablation (SMD = -1.93, 95% CI: -12.13~-1.73). The pooled results indicated that volume reduction rate (VRR) after thermal ablation at 3-year follow-up was 98.91% (95% CI: 97.98–99.83%), and complete disappearance rate (CDR) after thermal ablation at 3-year follow-up was 83% (95% CI: 67–94%). In addition, the incidence of newly discovered mPTC and lymph node metastases after thermal ablation was 0.3% (95% CI: 0.0–1.0%) and 0.0% (95% CI: 0.0–0.0%), respectively.

**Conclusion:**

Overall, the long-term (≥3 years) efficacy of ultrasound-guided thermal ablation in the treatment of PTC was significant, with favorable disease progression. Ultrasound-guided thermal ablation can be considered an alternative approach for patients with PTC who refuse surgery or are unable to undergo surgery.

## Introduction

Thyroid nodules are a common disease of the endocrine system; most are benign, and malignant nodules account for 5–15%. The most common type of malignant thyroid tumor is papillary thyroid carcinoma (PTC), accounting for 85% of all malignant thyroid tumors (1). It is associated with a favorable prognosis and a low mortality rate (2, 3). In PTC, if the diameter of the tumor is ≤1 cm, then it can be defined as papillary thyroid microcarcinoma (PTMC), which account for 50–60% of all PTC cases. Yu et al. reported 10- and 15-year cause-specific survival (CSS) rates for PTMC of 99.5% and 99.3%, respectively (4). Although surgery is the standard treatment for PTC, there is considerable disagreement regarding the management of PTC (5, 6). In general, the risk of PTC is very low, and the disease course is slow. Therefore, surgical thyroidectomy may be considered a very aggressive treatment option for some patients (6, 7). However, some patients are not candidates for surgery or are reluctant to undergo surgery for cosmetic reasons; therefore, exploration of other treatment options for PTC is imperative.

As a new minimally invasive treatment modality, ultrasound-guided thermal ablation, including laser ablation, radiofrequency ablation (RFA), and microwave ablation (MWA), has been reported in numerous attempts at treating PTC (8–12). In the past, surgery to treat papillary thyroid microcarcinoma did suffer from overtreatment. Therefore, many international counterparts suggested dynamic observation of PTC. Indeed, most patients with PTC did not show significant changes in dynamic observation. However, some patients withdrew from dynamic observation due to concerns or local progression and went for surgery instead (13). Thermal ablation therapy is the third option in addition to surgery and dynamic observation because it can effectively treat PTC without the huge trauma like surgery, nor will it cause obvious damage to thyroid function. Therefore, patients who are unwilling to perform dynamic observation in the past can use thermal ablation therapy, which can not only relieve the patient’s physical and psychological discomfort, but also avoid the damage caused by the operation, making thermal ablation therapy possible solution to solve the overtreatment of PTMC (13). These thermal ablation techniques all have unique advantages and have been shown to achieve favorable clinical results, with a low incidence of complications (14, 15). However, to date, there have been no evidence-based results addressing the long-term efficacy of thermal ablation applications in PTC. Therefore, we conducted a meta-analysis based on evidence-based medicine to explore the long-term (≥3 years) efficacy of thermal ablation in the treatment of PTC.

## Methods

### Literature inclusion and exclusion criteria

The inclusion criteria:

1) Study object: patients with PTC
2) Intervention measures: thermal ablation
3) Outcome indicators: volume of tumor, volume reduction rate (VRR), complete disappearance rate (CDR), incidence of newly discovered PTC, and lymph node metastasis (LNM)
4) Study design: non-randomized controlled trial (non-RCT)

The exclusion criteria: repeated publications, studies without full text or that could not conduct data extraction, studies using animal experiments, reviews, and systematic reviews.

### Search strategy

In this meta-analysis, we searched the PubMed, Embase, and Cochrane Library databases from establishment of the database to September 2022. The search terms were: (“Thyroid Cancer, Papillary” OR “Papillary Thyroid Cancer” OR “Papillary Thyroid Carcinoma” OR “Nonmedullary Thyroid Carcinomas” OR “papillary thyroid microcarcinoma” OR “PTMC”) AND (“radiofrequency ablation” OR “RFA” OR “laser ablation” OR “LA” OR “microwave ablation” OR “MWA” OR “thermal ablation”).

### Literature screening and data extraction

Two researchers independently performed the literature search, screening, and information extraction. When a question or dispute arose, we reached a consensus after discussion or negotiation. The data extraction included: author(s); article publication year; country; research type; number of patients; and outcome indicators, including tumor volume, VRR, CDR, incidence of newly discovered PTC, and LNM.

### Literature quality assessment

Two independent researchers assessed the quality of evidence for each study using the methodological index for non-randomized studies (MINORS) scale (16). There were 12 items in total, each with a potential score of 0 to 2 and a total score of 24. Based on score, the studies were classified as “moderate quality” from 9 to 16 and \ “high quality” from 17 to 24.

### Data synthesis and statistical analysis

We analyzed all data using STATA (version 15.1). Standardized mean difference (SMD) was used to assess differences in continuous variables. We used I^2^ and Q tests to evaluate heterogeneity. If the heterogeneity test was P ≥ 0.1 and I^2^ ≤ 50%, there was homogeneity among studies, and the fixed effects model was used for combined analysis; if P < 0.1 and I^2^ > 50%, there was heterogeneity, and a sensitivity analysis was conducted to identify its source. If the heterogeneity remained large, we used a random effects model or abandoned the combination of results and used descriptive analysis. We used a funnel plot and Egger’s test to assess publication bias.

## Results

### Results of the literature search

**Figure 1:**
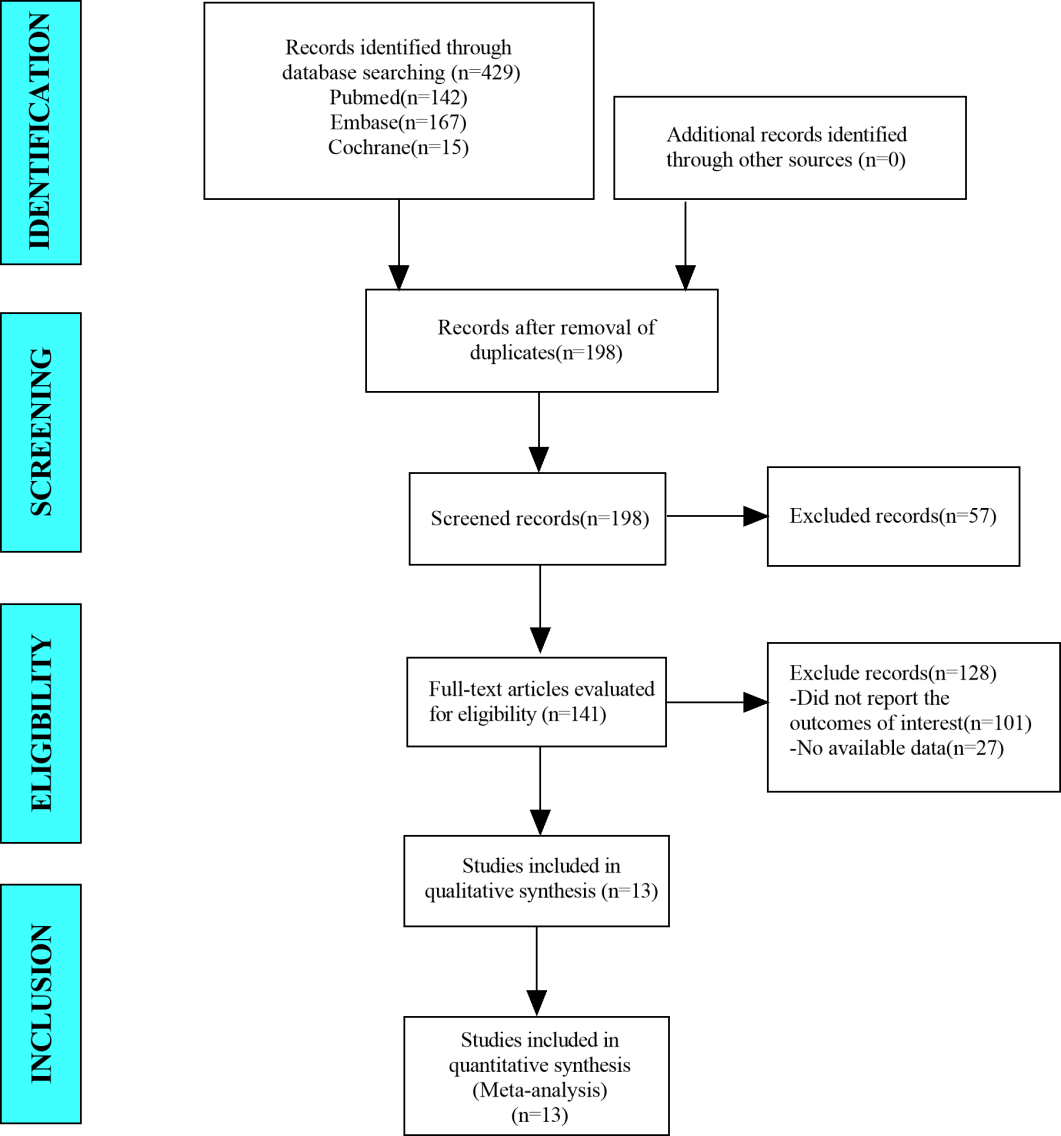
Flow diagram for selection of studies.

In this meta-analysis, we retrieved a total of 429 studies from the databases, which included PubMed, Embase, and Cochrane Library. After eliminating duplicate studies, 198 studies remained. After browsing titles and abstracts, 141 studies remained. After browsing full-text studies, we obtained 13 studies and excluded any that did not report the outcomes of interest or other ablation methods. Finally, we included 13 articles in the meta-analysis (**Figure 1**).

### Baseline characteristics and quality assessment of the included studies

In total, we included 13 non-RCT studies in this meta-analysis, and all were retrospective studies. The sample size was 2567 patients, and the number of tumors was 2572. MINORS scores were all above 16 points. The mean age distribution of patients was between 41.2 and 48.0 years, which is comparable. Thermal ablation modalities used in selected studies included MWA and RFA. Two studies received a score of 16, four studies scored 17, five studies scored 18, and two studies scored 19, indicating that the literature included was of moderate or high quality (**Table 1**).

**Table 1:** Baseline characteristics and quality assessment of the included studies.

Study	Country	Research type	No. of patients	Age	Gender (Female/male)	NO. of tumors	Volume of ablation area (mm3)	Ablation modalities	MINORS score
Teng et al., 2018 (17)	China	retrospective	15	48.0 ± 8.8	9/6	21	174.0 ± 259.1	MWA	16
Teng et al., 2020 (18)	China	retrospective	41	46.1 ± 8.9	/	41	55.8 (21.5-112.2)	MWA	18
Yan et al., 2020 (19)	China	retrospective	414	43.6 ± 9.8	323/91	414	92.7 ± 83.4	RFA	16
Cao et al., 2021a (20)	China	retrospective	725	46.0 ± 11.0	573/152	725	110.7 ± 90.6	MWA and RFA	17
Cao et al., 2021b (21)	China	retrospective	172	46.0 ± 13.0	38/134	172	810.6 ± 646.5	MWA and RFA	18
Wu et al., 2021 (22)	China	retrospective	106	44.4 ± 11.1	87/19	106	200.0 ± 300.0	MWA	17
Xiao et al., 2021 (23)	China	retrospective	131	41.2 ± 10.9	104/27	131	140.0 ± 100.0	RFA	18
Yan et al., 2021 (24)	China	retrospective	47	43.4 ± 9.3	34/10	100	75.2 ± 73.9	RFA	19
Zhu et al., 2021 (25)	China	retrospective	102	43.0 ± 19.0	82/20	102	60.0 ± 90.0	RFA	18
Zu et al., 2021 (26)	China	retrospective	320	45.0 ± 10.6	237/83	320	85.0 ± 89.1	MWA	18
Wei et al., 2022 (27)	China	retrospective	404	43.0 ± 12.0	289/115	350	200.0 ± 300.0	MWA	17
Wu et al., 2022 (28)	China	retrospective	69	45.6 ± 11.8	51/18	69	260.0 ± 350.0	MWA	17
Zheng et al., 2022 (29)	China	retrospective	21	45.1 ± 9.2	12/9	21	1390.0 ± 760.0	MWA	19

### Results of the meta-analysis

#### Tumor volume

**Figure 2:**
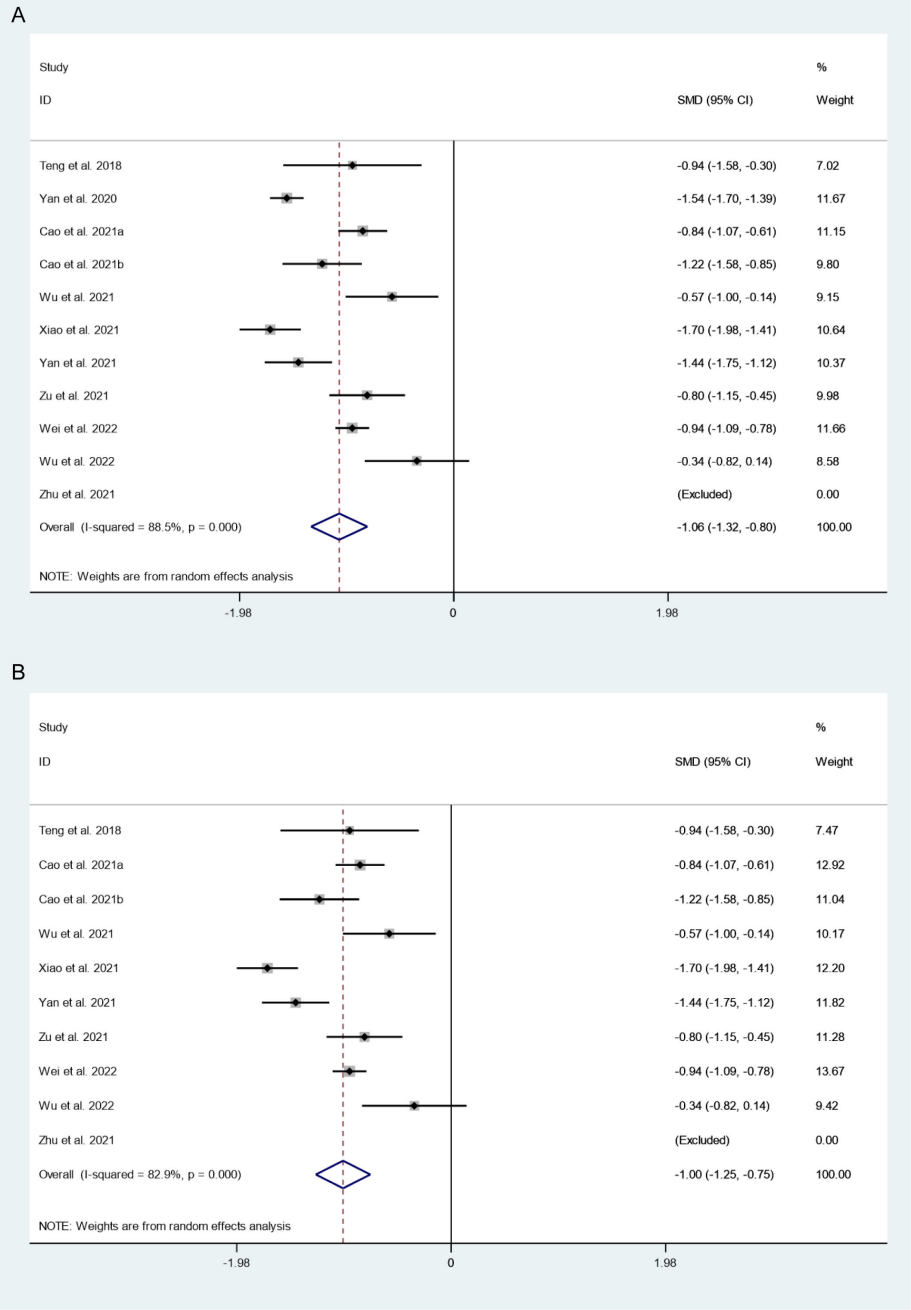
Changes in tumor volume after thermal ablation at 3-year follow-up **(A)** Before sensitivity analysis; **(B)** After sensitivity analysis).

There were 11 studies in which 2510 patients were enrolled that reported changes in tumor volume after thermal ablation at 3-year follow-up. Because there was significant heterogeneity (*I^2^ = 88.5%, P = 0.000*), we conducted a sensitivity analysis and found that the Yan et al.’s 2020 study had a large impact on the results (**Figure S1**). Heterogeneity decreased after excluding this study (*I^2^ = 82.9%, P = 0.000*) (**Figure 2A**), and we conducted a meta-analysis using a random effects model. The pooled results showed that tumor volume after thermal ablation at 3-year follow-up was significantly lower than pre-ablation (SMD = -1.06, 95% CI: -1.32~-0.80, P = 0.000; **Figure 2B**).

#### Maximum diameter

**Figure 3:**
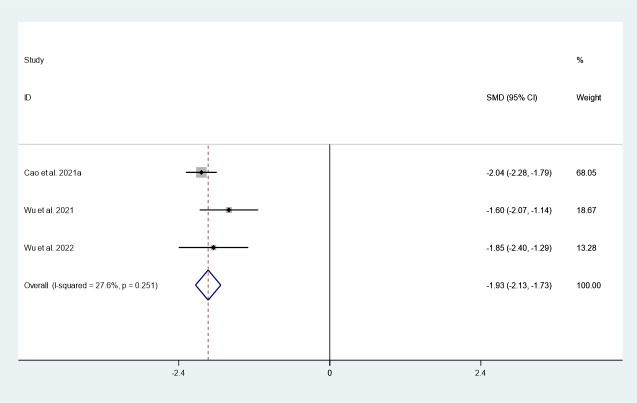
Changes in maximum diameter after thermal ablation at 3-year follow-up.

There were 3 studies in which 1036 patients were enrolled that reported changes in maximum diameter after thermal ablation at 3-year follow-up. Because there was no significant heterogeneity (*I^2^ = 27.6%, P = 0.251*), we conducted a meta-analysis using a fixed effects model. The pooled results indicated that the maximum diameter after thermal ablation at 3-year follow-up was significantly lower than pre-ablation (SMD = -1.93, 95% CI: -12.13~-1.73, P = 0.000; **Figure 3**).

#### VRR

**Figure 4:**
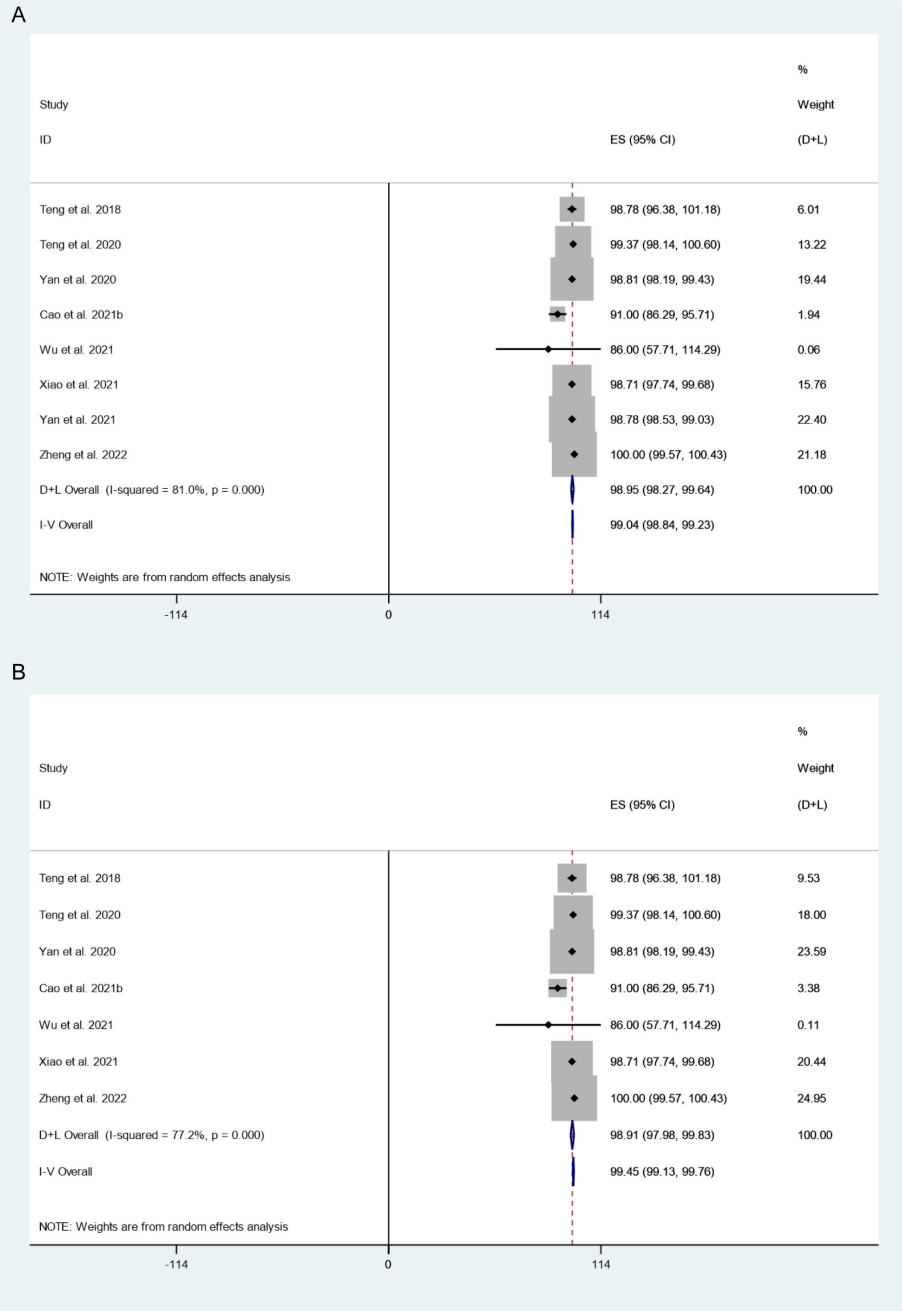
VRR after thermal ablation at 3-year follow-up **(A)** Before sensitivity analysis; **(B)** After sensitivity analysis).

There were 8 studies in which 794 patients were enrolled that reported VRR after thermal ablation at 3-year follow-up. Because there was significant heterogeneity (*I^2^ = 81.0%, P = 0.000*), we conducted sensitivity analysis and found that the Yan et al.’s 2021 study had a large impact on the results (**Figure S3**). Heterogeneity decreased after excluding this study (*I^2^ = 77.2%, P = 0.000*) (**Figure 4A**), and we conducted a meta-analysis using a random effects model. The pooled results indicated that VRR after thermal ablation at 3-year follow-up was 98.91% (95% CI: 97.98–99.83%; **Figure 4B**).

#### CDR

**Figure 5:**
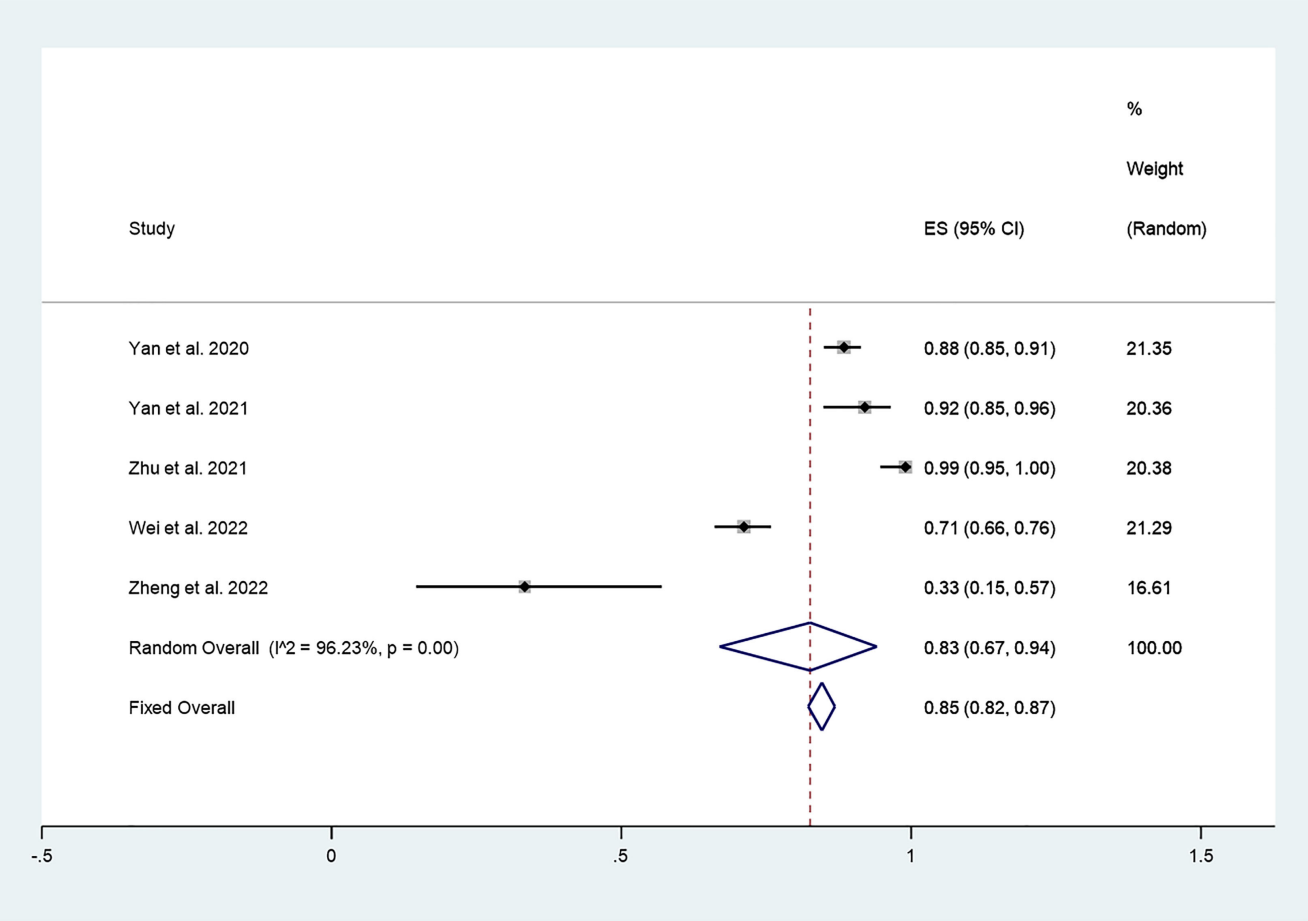
CDR after thermal ablation at 3-years follow-up.

There were 5 studies in which 987 patients were enrolled that reported CDR after thermal ablation at 3-year follow-up. Because there was significant heterogeneity (*I^2^ = 96.23%, P = 0.000*), we conducted a meta-analysis using a random effects model. The pooled results indicated that CDR after thermal ablation at 3-year follow-up was 83% (95% CI: 67–94%; **Figure 5**).

#### Incidence of newly discovered PTC

**Figure 6:**
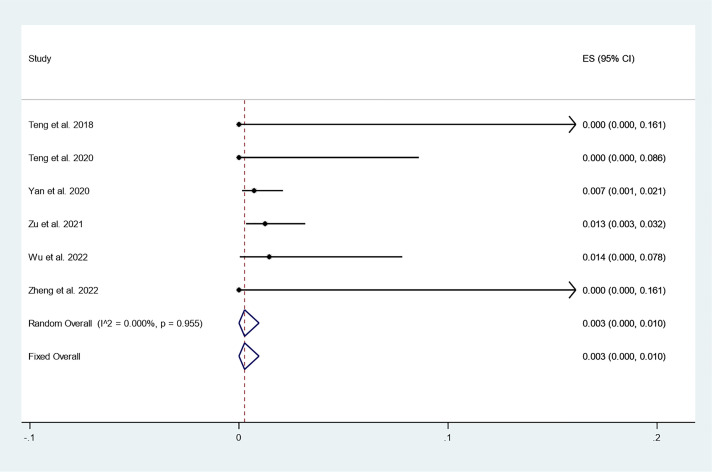
Incidence of newly discovered PTC after thermal ablation at 3-year follow-up.

There were 6 studies in which 886 patients were enrolled that reported the incidence of newly discovered PTC after thermal ablation at 3-year follow-up. Because there was no significant heterogeneity (*I^2^ = 0.000%, P = 0.955*), we conducted a meta-analysis using a fixed effects model. The pooled results indicated that the incidence of newly discovered PTC after thermal ablation at 3-year follow-up was 0.3% (95% CI: 0.0–1.0%; **Figure 6**).

#### Incidence of LNM

**Figure 7:**
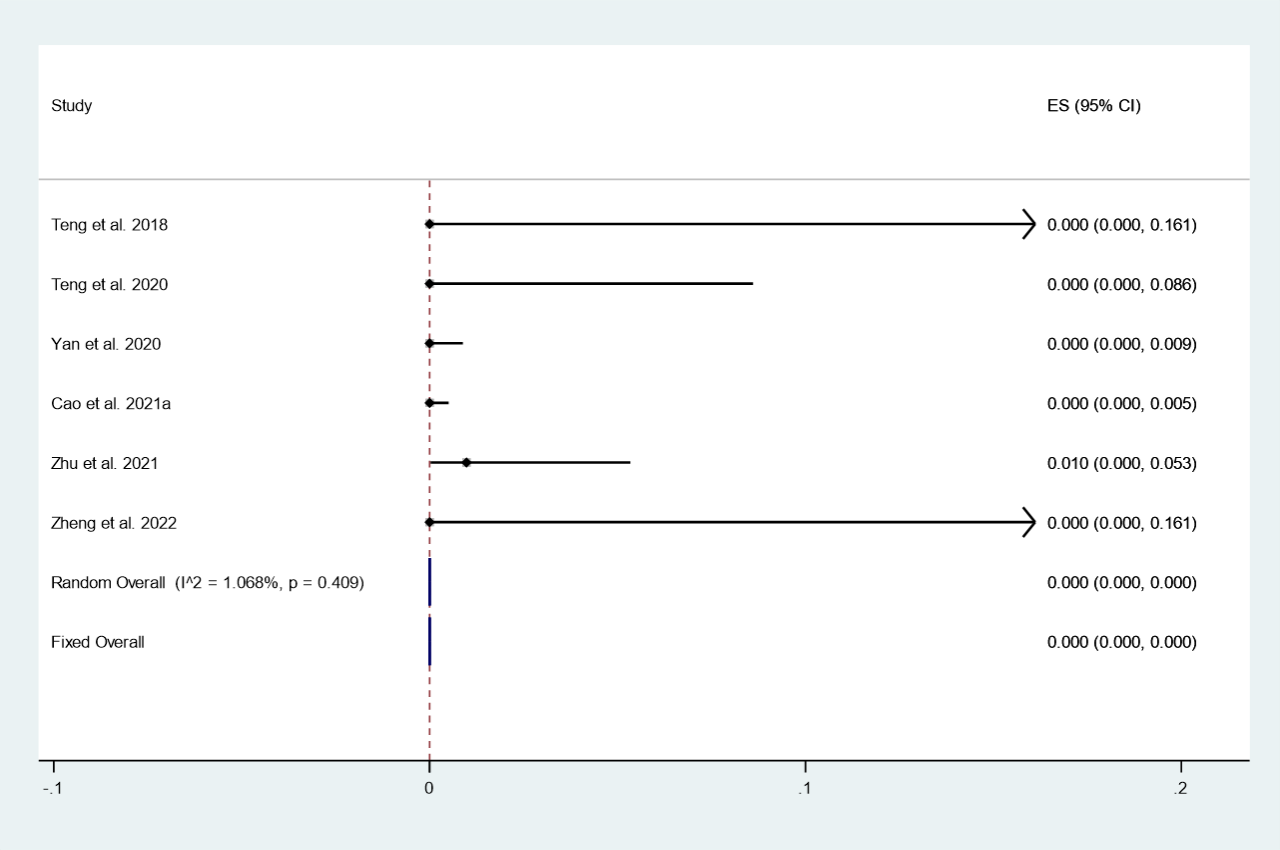
Incidence of LNM after thermal ablation at 3-year follow-up.

There were 6 studies in which 1324 patients were enrolled that reported the incidence of LNM after thermal ablation at 3-year follow-up. Because there was no significant heterogeneity (*I^2^ = 1.068%, P = 0.409*), we conducted a meta-analysis using a fixed effects model. The pooled results indicated that the incidence of LNM after thermal ablation at 3-year follow-up was 0.0% (95% CI: 0.0–0.0%; **Figure 7**).

### Subgroup analysis

To further explore the differences in the efficacy of different thermal ablation modalities, we conducted subgroup analysis.

#### Tumor volume

**Figure 8:**
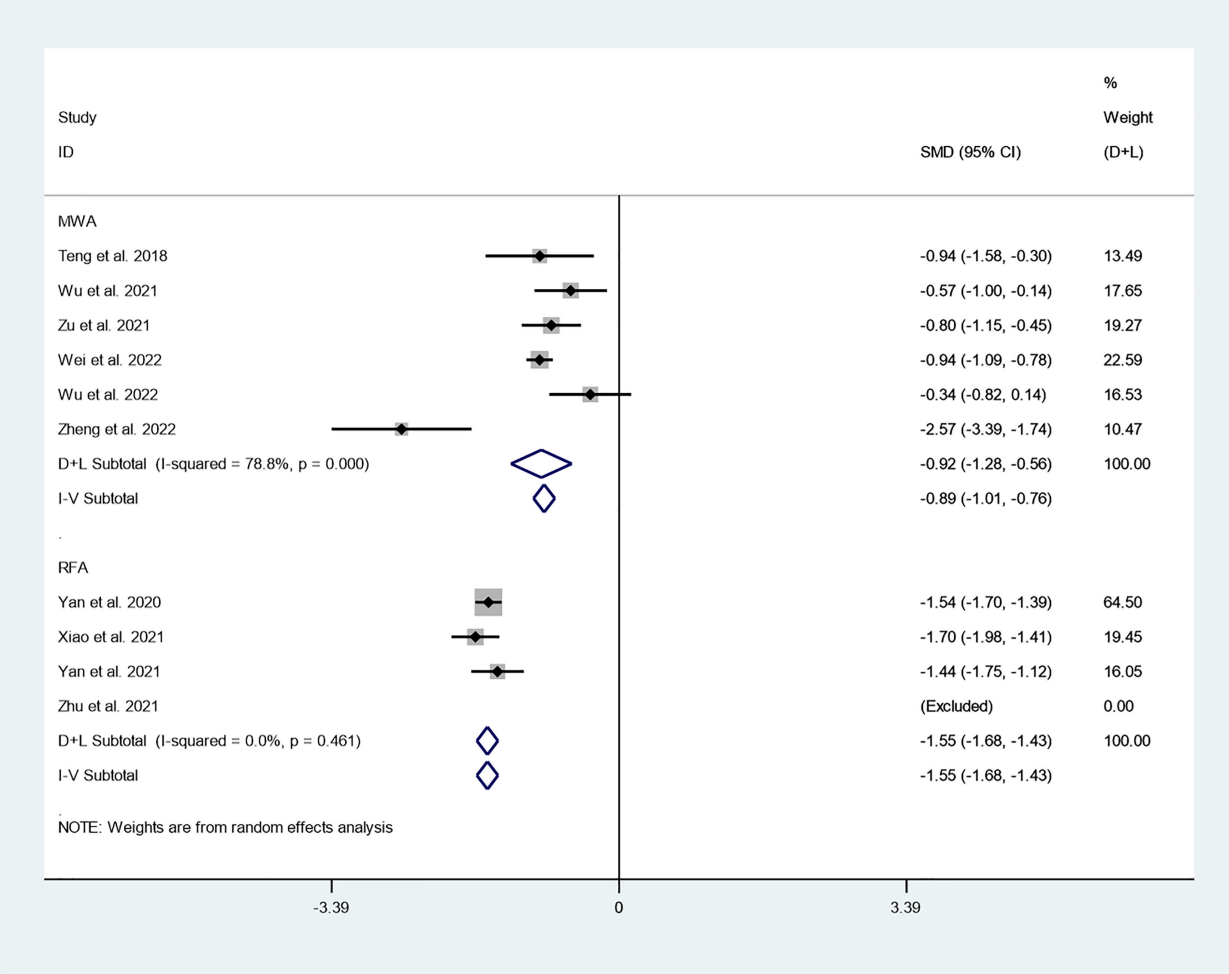
Changes in tumor volume after different thermal ablation modalities at 3-year follow-up.

We first explored differences in the effect of different thermal ablation modalities on changes in tumor volume through subgroup analysis. There were 6 studies that reported changes in tumor volume after MWA at 3-year follow-up. Because there was significant heterogeneity (*I^2^ = 78.8%, P = 0.000*), we conducted a meta-analysis using a random effects model. The pooled results indicated that tumor volume after MWA at 3-year follow-up was significantly lower than pre-ablation (SMD = -0.92, 95% CI: -1.28~-0.56, P = 0.000; **Figure 8**). There were six studies that reported changes in tumor volume after RFA at 3-year follow-up. Because there was no significant heterogeneity (*I^2^ = 0.0%, P = 0.461*), we conducted a meta-analysis using a fixed effects model. The pooled results indicated that tumor volume after RFA at 3-year follow-up was significantly lower than pre-ablation (SMD = -1.55, 95% CI: -1.68~-1.43, P = 0.000; **Figure 8**).

#### VRR

**Figure 9:**
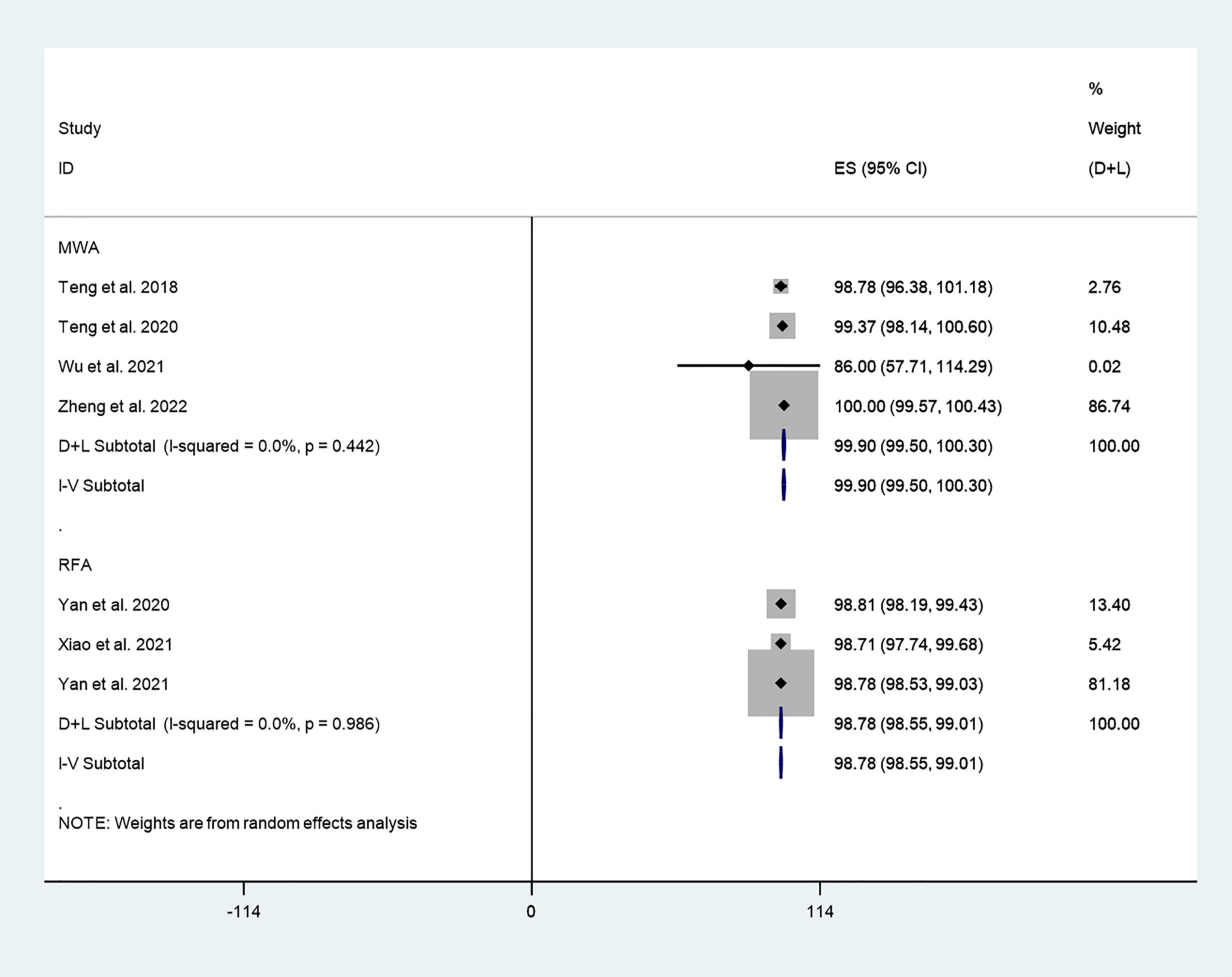
VRR after different thermal ablation modalities at 3-year follow-up.

We explored differences in the effect of different thermal ablation modalities on VRR through subgroup analysis. The pooled results indicated that VRR after MWA at 3-year follow-up was 99.90% (95% CI: 99.50–100.30%; *I^2^ = 0.0%, P = 0.442*; enrolling 4 studies; **Figure 8**). In addition, the pooled results indicated that VRR after RFA at 3-year follow-up was 98.78% (95% CI: 98.55–99.01%; *I^2^ = 0.0%, P = 0.986*; enrolling 3 studies; **Figure 9**).

#### CDR

**Figure 10:**
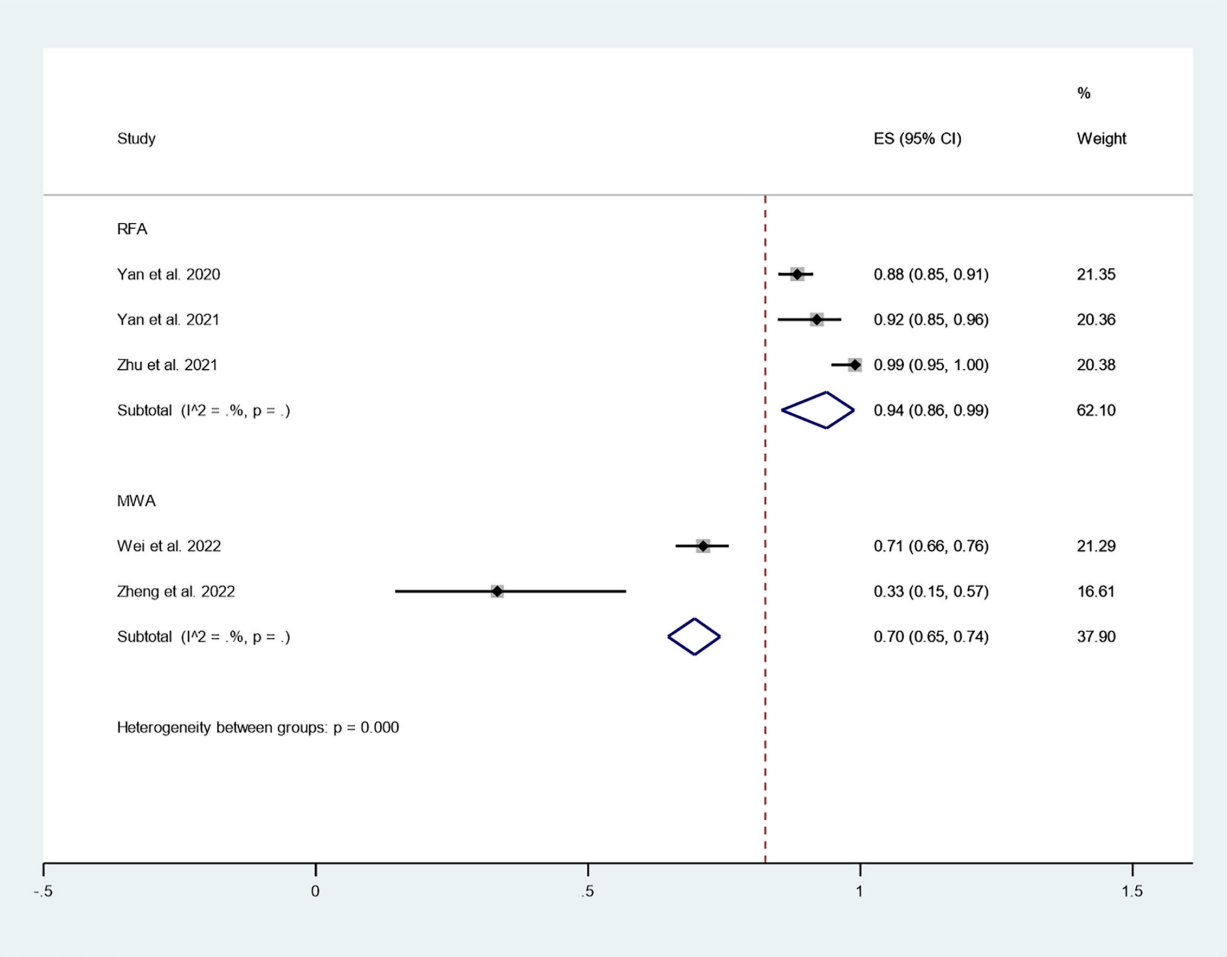
CDR after different thermal ablation modalities at 3-year follow-up.

Furthermore, we explored differences in the effect of different thermal ablation modalities on CDR through subgroup analysis. The pooled results indicated that CDR after RFA at 3-year follow-up was 94% (95% CI: 86–99%; enrolling 3 studies; **Figure 9**). In addition, the pooled results indicated that CDR after MWA at 3-year follow-up was 70% (95% CI: 65–74%; enrolling 2 studies; **Figure 10**), and the CDR after 3 years of RFA treatment of PTC was significantly higher than MWA (P=0.000; **Figure 10**).

### Sensitivity analysis

We conducted sensitivity analysis by eliminating each included study one by one and performing a summary analysis of the remaining studies. The results of the sensitivity analysis are presented in **Figures S1–S5**.

### Publication bias

**Figure 11:**
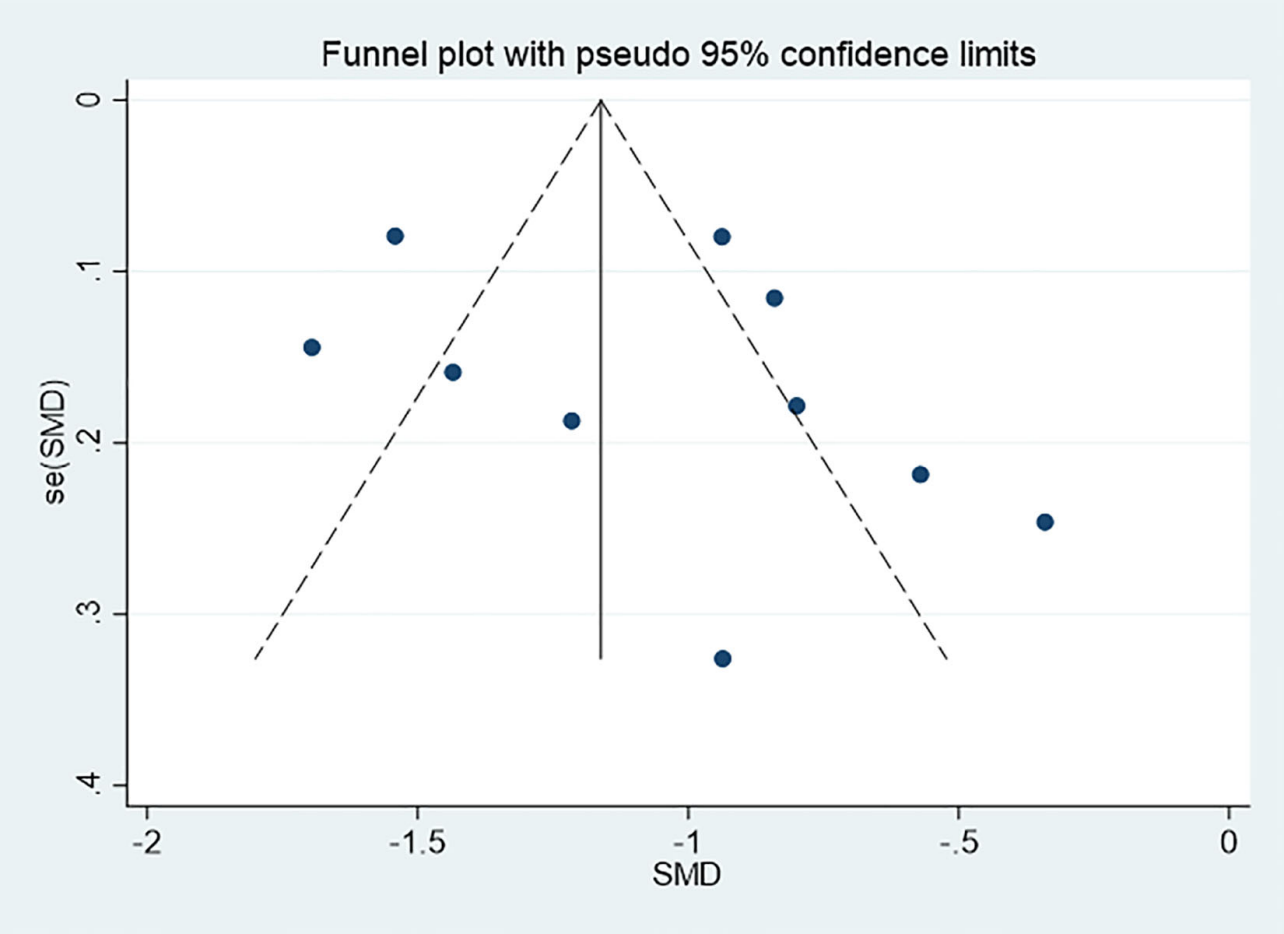
Funnel plot for assessing publication bias.

The funnel plot is shown in **Figure 11**. The funnel plot was symmetrical, and the P value of Egger’s test was 0.352, indicating that there was no obvious publication bias in this study.

## Discussion

This meta-analysis analyzed the efficacy of thermal ablation for PTC by pooling 13 studies, including 2567 patients, that reported on the application of ultrasound-guided thermal ablation in PTC to provide guidance for clinical treatment.

Because PTC develops gradually, long-term follow-up is necessary to assess the effectiveness of thermal ablation. Our pooled results indicated that RFA treatment significantly reduced tumor volume and maximum diameter, with a VRR and CDR of 98.91% (95% CI: 97.98–99.83%) and 83% (95% CI: 67–94%) at 3 years after thermal ablation, respectively. These results suggest that thermal ablation can be considered an alternative approach in patients with PTC who refuse surgery or are unable to undergo surgery. All included studies involved ultrasound, which may be due to its ability to accurately assess the extent of the ablation area and the treatment effect (30).

To further explore the differences in the efficacy of different thermal ablation modalities, we conducted subgroup analysis. First, we found that within-group heterogeneity still existed in the MWA subgroup (*I^2^ = 78.8%, P = 0.000*); however, the heterogeneity was lower than in the analysis of all thermal ablation modalities (*I^2^ = 88.5%, P = 0.000*). In addition, there was no significant heterogeneity in the RFA subgroup (*I^2^ = 0.0%, P = 0.461*). This suggests that differences in ablation methods may be one of the reasons for the heterogeneity. The pooled results showed that tumor volume after MWA and RFA at 3-year follow-up was significantly lower than pre-ablation. These results suggest that either MWA or RFA can be used as an alternative to surgery. We then explored differences in the effect of different thermal ablation modalities on VRR *via* subgroup analysis. Likewise, we found no significant heterogeneity in either MWA or RFA subgroups. The pooled results indicated that VRR after MWA at 3-year follow-up was 99.90% (95% CI: 99.50–100.30%), and VRR after RFA at 3-year follow-up was 98.78% (95% CI: 98.55–99.01%). VRR in both cases was close to 100%. Despite the lack of statistical analysis, it is clear that the VRRs of the two treatments are very similar. In addition, the pooled results showed that CDR after RFA and MWA at 3-year follow-up was 94% (95% CI: 86–99%) and 70% (95% CI: 65–74%), respectively. Furthermore, CDR after 3 years of RFA treatment of PTC was significantly higher than that of MWA (P=0.000). This suggests that RFA may have better long-term efficacy than MWA.

In addition, we focused on tumor progression, including newly discovered PTC and LNM. The pooled results indicated that the incidence of newly discovered PTC after thermal ablation at 3-year follow-up was 0.3% (95% CI: 0.0–1.0%). In addition, the pooled data indicated that the incidence of LNM after thermal ablation at 3-year follow-up was 0.0% (95% CI: 0.0–0.0%). The reported incidence of newly discovered PTC and LNM after surgery was 3.2% and 0.7%, respectively (27). In addition, Zu et al. (26) reported that the incidence of LNM after surgery for PTC was 2.5%. This suggests that long-term disease progression after thermal ablation for PTC may have better prospects than surgery.

This study has several limitations. First, in the analysis of tumor volume, although our subgroup analysis for ablation modalities determined that differences in ablation modalities may be one of the reasons for the heterogeneity, we could not completely rule out the intragroup heterogeneity of the MWA subgroup. This should be explored further in the future. In addition, different teams used different ablation modalities, and the technical proficiency of operators may also have been a source of heterogeneity. Second, the studies included in this meta-analysis were all retrospective studies, which is due to the limitations of the current research. More prospective studies are needed to corroborate our findings in the future.

## Conclusion

Overall, the long-term efficacy (≥3 years) of ultrasound-guided thermal ablation in the treatment of PTC was significant, with favorable disease progression. Ultrasound-guided thermal ablation can be considered an alternative approach for patients with PTC who refuse surgery or are unable to undergo surgery.

## Data availability statement

The original contributions presented in the study are included in the article/**Supplementary Material**. Further inquiries can be directed to the corresponding authors.

## Author contributions

JX collected data and wrote the manuscript, DT and HW conceived the manuscript. All authors have read and approved the final manuscript.

## Funding

The study was supported by Jilin Provincial Health and Family Planning Commission (2019SCZ027) and (2020SCZ08).

## Conflict of interest

The authors declare that the research was conducted in the absence of any commercial or financial relationships that could be construed as a potential conflict of interest.

## Publisher’s note

All claims expressed in this article are solely those of the authors and do not necessarily represent those of their affiliated organizations, or those of the publisher, the editors and the reviewers. Any product that may be evaluated in this article, or claim that may be made by its manufacturer, is not guaranteed or endorsed by the publisher.
