# Supplementary material for: Over than three-year follow-up results of thermal ablation for papillary thyroid carcinoma: A systematic review and meta-analysis
Source: Front Endocrinol (Lausanne). 2022 Oct 24;13:971038. doi: 10.3389/fendo.2022.971038 (PMC9637737; doi:10.3389/fendo.2022.971038)
Supplement: Supplementary file 1 [file DataSheet_1.docx]

Figure S1. Sensitivity analysis of the changes in volume of tumors after thermal ablation at 3-years follow-up

Figure S2. Sensitivity analysis of the changes in maximum diameter after thermal ablation at 3-years follow-up

Figure S3.Sensitivity analysis of the VRR in volume of tumors after thermal ablation at 3-years follow-up

Figure S4. Sensitivity analysis of the CDR in volume of tumors after thermal ablation at 3-years follow-up

Figure S5. Sensitivity analysis of the incidence of newly discovered PTC after thermal ablation at 3-years follow-up

Figure S6. Sensitivity analysis of the incidence of LNM after thermal ablation at 3-years follow-up
